# Fairness norm violations in anti-social psychopathic offenders in a repeated trust game

**DOI:** 10.1038/s41398-019-0606-3

**Published:** 2019-10-21

**Authors:** Lisa A. Rosenberger, Daniela M. Pfabigan, Benjamin Lehner, Katinka Keckeis, Eva-Maria Seidel, Christoph Eisenegger, Claus Lamm

**Affiliations:** 1 Neuropsychopharmacology and Biopsychology Unit, Department of Basic Psychological Research and Research Methods, Faculty of Psychology, University of Vienna, Vienna, Austria; 2 Social, Cognitive and Affective Neuroscience Unit, Department of Basic Psychological Research and Research Methods, Faculty of Psychology, University of Vienna, Vienna, Austria; 3 Faculty of Medicine, Institute of Basic Medical Sciences, Department of Behavioural Sciences in Medicine, University of Oslo, Oslo, Norway; 4 Prison for Mentally Disordered Offenders (Justizanstalt Wien Mittersteig), Vienna, Austria

**Keywords:** Psychiatric disorders, Physiology, Human behaviour

## Abstract

Psychopathic offenders have a high propensity to violate social norms, as indicated for instance by their widespread lying and cheating behaviour. The reasons for their norm violations are not well understood, though, as they are able to recognise norms in a given situation and also punish norm violators. In this study, we investigated whether psychopathic offenders would violate fairness norms during a repeated trust game because of increased profit-maximising concerns. We measured back-transfer decisions in the repeated trust game, and affective arousal by means of skin conductance responses (SCR) in violent offenders with varying degrees of psychopathy, and non-offenders with low-trait psychopathy. Psychopathy in offenders was measured with the Psychopathy Checklist–Revised (PCL-R). In the task, a participant and an interaction partner entrusted each other money for multiple rounds with the goal to earn as much money as possible. Fairness norm violations were positively associated with Factor 2 scores (the lifestyle/anti-social psychopathy subscale) of the PCL-R, but this was not accompanied by clear profit-maximising behaviour. In addition, anticipatory arousal to self-advantageous decisions was higher in all offenders, independent of their degree of psychopathy, compared with non-offenders. The results of our study widen our understanding of social decision-making in psychopathy. They also suggest treatment possibilities in offenders scoring high on Factor 2, targeting empathic concern and related prosocial intentions to overcome norm-violating behaviour.

## Introduction

Adhering to social norms is an integral part of successful functioning in everyday life. They help coordinate behaviour between people in situations that carry a conflict between self-interest and cooperative outcomes^1^. Norm adherence usually is prosocial and increases outcomes of the group, even though this often comes at the expense of one’s own profits^2^. However, not all individuals are willing to adhere to social norms to the same extent. For example, psychopathic individuals have a reputation of violating social norms by lying, cheating and deceiving on a regular basis^3^. They are also highly reward sensitive^4–6^, which is why they might favour their own over group profits, and hence neglect social norms. This implies, though, that they should build a negative reputation and function very poorly in our society, but many studies on psychopathic personality traits in corporate environments suggest the opposite^7^. We aimed to shed light on this contradiction by studying social norm adherence and underlying affective arousal in offenders with varying degrees of psychopathy. Affective arousal was assessed by means of skin conductance responses (SCR), and fairness norm violations were assessed in a behavioural economics approach, using a repeated trust game. This approach is unique, as most previous decision-making research on psychopathy had used single-shot games, whose limitations could be overcome by the repeated nature of our task design. Most importantly, this enabled us to disentangle behaviour driven by profit-maximising self-interest from group-benefiting norm adherence.

To adhere to norms, it is important to recognise a shared norm in a given situation, as well as a deviation from it^8^. The large majority of individuals not only expects others to adhere to these norms but also punishes others if they do not do so^1^, even if this comes at costs to the self^9^. Psychopathic offenders are able to understand social norms in two player decision-making paradigms: they punish norm violators in an ultimatum game as often^10^, or even more often than healthy control participants^11^. However, much less is known about psychopathic offenders’ norm adherence in these contexts. Evidence from single-shot decision-making studies—in which two people interact with each other exactly once and reputation effects do not play a role—suggest that even though psychopathic offenders behave highly profit-maximising, they seem to adhere to fairness norms when there is the threat of retaliation from the interaction partner. In a dictator game, where the interaction partner has no opportunity to respond with retaliation or other ways of punishment of unfair offers, psychopathic offenders behave in their own self-interest and keep most of an allocated amount of money to themselves^11–13^. In contrast, in the ultimatum game, an interaction partner gets the opportunity to reject an unfair offer. Here, psychopathic offenders favour joint outcomes and thus adhere to the norm of the game by splitting their allocated money in the same fair way as non-psychopathic participants^11,12^. To our knowledge, only one study investigated psychopathic offenders’ behaviour during repeated decision-making interactions^14^. Paradigms where participants interact with each other multiple times resemble real-world situations more closely than single-shot versions, as decisions are not taken in isolation but are informed by the possible consequences of the interaction partner’s extended behaviour. These repeated interactions can either be simultaneous, or sequential in nature. In a repeated prisoner’s dilemma, where two players decide simultaneously whether they want to cooperate with each other, psychopathic offenders manipulated their partners into believing they would adhere to a cooperative norm. In reality they betrayed them, maximised their own outcomes and at the end earned more money than their partners^14^. Thus, decision-making studies have shown so far that, even though psychopathic offenders expect others to adhere to norms that maximise group profits and punish them if they do not do so, they do not seem to adhere to them themselves in simultaneous decision-making paradigms when there is no clear threat of retaliation.

Oftentimes, we do not make simultaneous decisions (as in Mokros and colleagues’ paradigm^14^), but rather react to our interaction partner in a sequential manner. In these situations, decisions are more easily interpreted as direct responses to the interaction partner’s behaviour, rather than an independent strategy. Negative behaviour gets punished immediately and can lead to a break down of cooperation or even of interactions altogether^15^. The repeated trust game is such a paradigm with which repeated sequential decision-making is studied^16^. In this task, two players (an investor and a trustee) make sequential decisions to entrust each other money during multiple interactions, which are rooted in different motivations. A decision can be informed by inequity aversion, which is a dislike for situations where either of the players is worse off^17^. In that case, a participant would adhere to a fairness norm that favours equal profits, and would therefore equalise the earnings of the investor and the trustee in a given round. From previous research, it is not clear whether psychopathic offenders’ behaviour would be driven by inequity aversion to the same extent as healthy participants during repeated sequential interactions. We address this by studying the psychopathic offender’s fairness norm adherence during a repeated trust game.

Decisions in a repeated trust game can also be rooted in monetary self-interest and the valuation of monetary rewards. Psychopathic offenders are known to process and react to rewards differently than non-psychopathic offenders. They have difficulties inhibiting their impulses when they can earn rewards^18^, and prefer immediate over delayed rewards^19^. In addition, there is an increased activation^6^ and a greater connectivity with frontal brain areas^4^ during reward processing in the ventral striatum of psychopathic offenders compared with healthy control subjects. Arnett^20^ described the motivational processes in psychopathic offenders as an imbalance between the punishment and reward system. Even though many studies have supported Arnett’s hypothesis of psychopathic offenders’ hyporesponsiveness to negative stimuli, Arnett’s prediction of hyperresponsive arousal to rewarding stimuli has only attracted little research with mixed findings, e.g., ref. ^21^. Even though economic decision-making paradigms lend themselves perfectly to test hyperresponsive arousal to rewards, we are not aware of any decision-making study that included arousal measures in psychopathic offenders. We addressed this in our study and hypothesised that psychopathic offenders’ aberrant reward processing would manifest itself both on a behavioural level, i.e., in terms of their decisions during the repeated trust game, and on the affective arousal level, as measured by SCR. On a behavioural level, we expected a preference for own rather than joint profits in psychopathic offenders, and therefore a neglect of the fairness norm. On the psychophysiological level, we expected the psychopathic offenders to display hyperresponsive arousal to both the anticipation and the receipt of rewards.

We tested these hypotheses by comparing fairness norm adherence behaviour during a repeated trust game in two groups: violent offenders with varying degrees of psychopathy, and a matched non-delinquent group scoring low on psychopathy. Within the offender group, we hypothesised that the effects would be mainly driven by Factor 1, which is the interpersonal and affective subscale of our psychopathy measure (Psychopathy Checklist–Revised (PCL-R)^3^. Factor 1 captures the deceptive and manipulative nature that is linked to norm violations. Participants played the task as trustees, i.e., as the person who could decide through their back-transfer whether or not to fully or partially reciprocate the trust invested in them.

## Methods

### Participants

We recruited 30 male incarcerated offenders from an Austrian prison (Wien Mittersteig), a facility for prisoners with a psychiatric disorder who are deemed criminally responsible according to the Austrian criminal code §21, para. 2. We excluded three participants with intelligence scores one standard deviation below the average of the offender sample, and one due to non-violent offences. One offender aborted the study after the first task (the task battery consisted of four tasks in total, findings for those outside the scope of this paper will be described elsewhere). Thus the final offender sample consisted of 25 male violent offenders. On average, the offenders were imprisoned for 4.58 years (SD = 3.8). Education levels of all offenders were limited to compulsory education level (i.e., 9 years of schooling in Austria, *N* = 7), vocational training level (*N* = 16) and secondary education level (*N* = 2). Eleven offenders were taking psychiatric medication at the date of testing, which included antipsychotics (*N* = 7), antidepressants (*N* = 2), a mix of the two (*N* = 1) or tranquilisers (*N* = 1). Importantly, the analyses reported below did not change depending on medication or duration of imprisonment^22^. Note that while the PCL-R total scores of our offender sample lies below the average of American cohorts (see ref. ^11^), they are comparable with other European samples (see refs. ^14,23,24^).

In addition, we recruited 28 male non-delinquent participants from a community cohort that were matched with the offender sample on age, education level and intelligence. Participants were recruited via public advertisements in local hospitals, supermarkets, job centres and through online postings on local job search sites. Participants were excluded because of missing skin conductance data (*N* = 2), psychopathy scores three times above the sample average (*N* = 1) and back-transfers of 0 MU (*N* = 2), which made it impossible to analyse behavioural variation. Thus the final non-delinquent group consisted of 23 male participants. Education levels of the non-offenders included compulsory education (*N* = 7), vocational training (*N* = 12) and secondary education (*N* = 4). None of the non-offenders had any history of psychiatric or neurological disorders or drug abuse (screened with the SCIDPIT light^25^ and the SCID II^26^). The study was approved by the local university ethics committee, as well as the institutional review board of the correctional treatment facility, and was conducted according to the declaration of Helsinki^27^. All participants provided informed consent before the start of the test session. Descriptives of age and questionnaire scores of all groups can be found in Table 1.

**Table 1:** Questionnaire scores and age of the two groups

	Offenders mean (range)	SD	Non-offenders mean (range)	SD	*t*-value	*p*-value
Age	35.41	10.19	34.65	10.12	−0.26	0.79
*PCL-R*						
Total	20.36 (8–32)	6.97				
Factor 1	9.2 (2–14)	3.07				
Factor 2	9.0 (1–16)	4.56				
*PPI-R*						
Total			294 (253–322)	17.83		
Factor 1			110.76	12.15		
Factor 2			151.32	17.75		
Coldheartedness		31.4	5.82			
BIS 11	53.96	9.3	62.13	9.56	3.00	<0.01
SPM	32.88	30.39	26.7	3.46	−1.01	0.32
ITS	71.92	13.81	74.26	11.56	0.64	0.53

### Questionnaires

To assess psychopathic personality traits, we used the Psychopathy Checklist–Revised (PCL-R)^3^ in the offenders, and the Psychopathic Personality Inventory–revised (PPI-R)^28^ in the non-offenders. High scores reflect a high degree of psychopathy. We used the continuous PCL-R scores to analyse the effect of psychopathy on norm violations in the offender sample. Impulsivity was measured with the Barratt Impulsiveness Scale (BIS-11)^29^. Intelligence was assessed with Raven’s Standard Progressive Matrices (SPM)^30^, and self-reported trait interpersonal trust with the Interpersonal Trust Scale (ITS)^31^. Details about the questionnaires can be found in the Supplementary Materials. Since these questionnaire scores were not the main focus of the study, we only included them as covariates of no-interest in all group comparison analyses to control for between-group variations.

### Task–repeated trust game

Participants performed the task on a Dell Latitude D630 laptop (Intel Core Dual 800 MHz, 14.100), and they were led to believe that they were connected through the internet with an interaction partner. The data collection was conducted by two male experimenters. One served as the experimenter, while the other one served as an interaction partner for the current task. This confederate was introduced as player B, without providing any additional information on his background. The two laptops were separated by a screen that prevented the participant and the confederate from seeing each other. Contact between the confederate and the participant was restricted to in-game interactions. Participants were explicitly instructed that they would not meet again after the test session and would not be able to interact with each other outside the task. The task was programmed in E-Prime 2.0^32^.

**Fig. 1:**
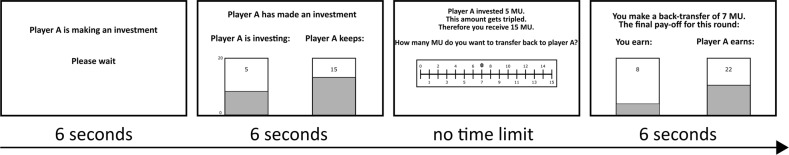
Time course of a single interaction in the repeated trust game including the translated screens (from German) presented to the participants

In the task, two players, playing as investor and trustee, interacted with each other for 20 rounds. Each round the investor got endowed with 20 monetary units (MU) and, as the first-mover, had to send any amount from his endowment to the trustee. This investment got automatically tripled by the experimenter and was sent to the trustee. The trustee, as the second-mover, had to make a back-transfer decision that could be any amount between 0 and the tripled investment. At the end of the round, both players were presented with the MU they earned in this round. The profits from each round were added up, exchanged to Euros (exchange rate: 1 MU = 2 Eurocent) and paid out at the end of the game. The exact timing of a trial is displayed in Fig. 1. Participants always played the role of trustee and were paired throughout the task with the same investor. Investments were preprogrammed (see Supplements for details) and resembled a tit-for-tat strategy based on investment decisions of healthy participants in King-Casas et al.^15,33^. The repeated trust game task was always administered at the end of the task battery (consisting of a dictator game, an ultimatum game and a promise-deception task), and results of the other three tasks will be reported elsewhere.

### Skin conductance responses (SCR)

SCR was measured with an eight-channel bioamplifier (Mobi8-BP; TMSI B. V., Enschede, The Netherlands) with a 24 -bit analogue-to-digital (A/D) conversion rate. A custom-specific skin conductance sensor, which consisted of two flat Ag/AgCl electrodes, was used to obtain unfiltered raw skin conductance data. Before applying the electrodes, participants had to wash their hands with curd soap to ensure a similar skin condition at the beginning of the measurement across participants. Electrodes were applied to the distal phalanges of the index and middle finger of the non-dominant hand. Portilab 2.0 software was used to acquire time-logged SCR. The data were preprocessed in MATLAB (version 7.9.0) via the Ledalab toolbox (V3.2.3). Raw skin conductance data were first filtered with a 1 Hz 4th order IIR Butterworth low-pass filter, and then downsampled from 1024 Hz to 10.24 Hz. Phasic SCR, depicting the average magnitude within a predefined time window, were extracted with a continuous decomposition analysis (CDA)^34^ using a threshold of 0.1 µS.

We measured SCRs at three time points during each trial, each with a duration of 6000 ms. *SCR investment* is measured from the onset of the investor’s investment decision screen. Statistics are not reported, as they are out of scope of this study. *SCR anticipation* is measured within 6 s before the participant made his back-transfer decision. In case latencies from *SCR investment* and *SCR anticipation* overlapped for >3000 ms (i.e., when the participant made a back-transfer decision quicker than 3000 ms), we excluded these trials from analyses. In the offender sample, these were 9.40% (47 out of 500 trials) of the trials. In the non-delinquent group, these were 6.30% (29 out of 460 trials) of the trials. These were 8.13% of the other-advantageous decision trials, 6.10% of the fair decision trials, and 8.81% of the self-advantageous decisions trials. Finally, we also measured *SCR outcome* from the onset of the outcome screen. We corrected the three extracted SCR measures for non-normal distribution with a log-transformation and performed an outlier correction based on boxplots, deleting datapoints that lie outside 1.5 times the interquartile range.

### Code availability

For research transparency, all our analysis scripts are published online^22^. We are not able to publish our data due to confidentiality concerns related to the highly specific prisoner sample.

### Statistical analyses

We analysed the raw data with (generalised) linear mixed models with orthogonal contrasts and always included the highest order within-participant predictor as a random slope^35^, and participant as a random intercept. We used return-ratios ( = $\frac{{back - transfer}}{{investment}}$) instead of the raw back-transfers in the models for an easier interpretation of the effects, since raw back-transfers cannot be easily interpreted as high or low, as they always depend on the investment size. Return-ratios <1 reflect back-transfers that were smaller than the investments, and return-ratios >1 reflect back-transfers that were higher than the investments. Since we were specifically interested in fairness violations, we categorised return-ratios into fair (back-transfer amount resulted in an equal share of overall profits for investor and participant in a given round, i.e., profit investor = participant), and unfair (i.e., profit investor ≠ participant) decisions. Unfair decisions were further split up into self-advantageous (profit participant > investor) and other-advantageous (profit participant < investor) decisions. For all behavioural and arousal analyses, we first compared offenders and non-offenders, and in case of a group difference we repeated the same analysis with only the offender sample to test for effects depending on Factor 1 and Factor 2 scores of the PCL-R. In the group comparisons, we included ITS, SPM and BIS 11 scores as covariates of no interest to control for variations in these scores between the groups. All tests were two-sided with an alpha of 0.05.

## Results

**Fig. 2:**
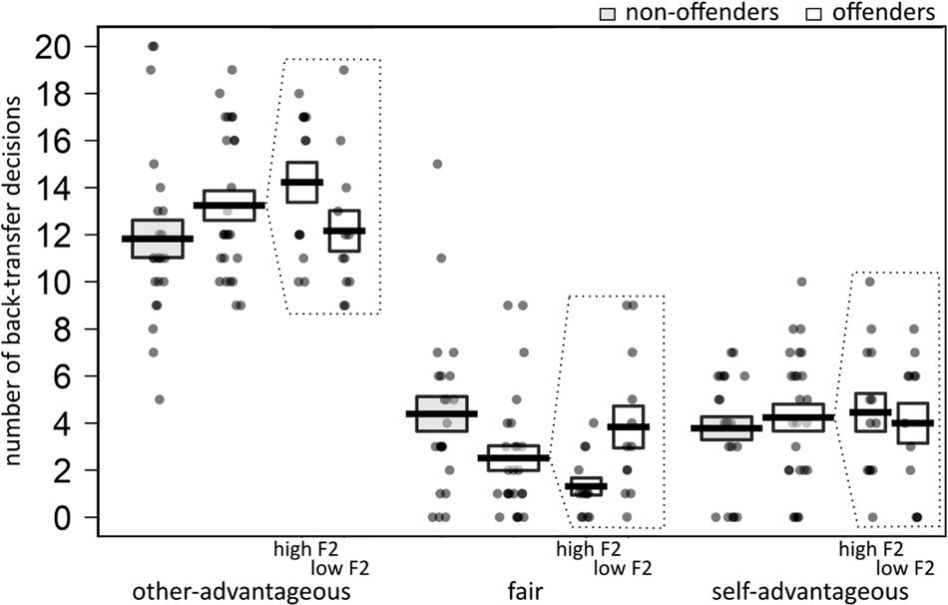
Offenders made less fair back-transfer decisions than non-offenders and this was negatively associated with Factor 2 scores. To visualise effects of Factor 2, we categorised offenders into high-scoring (high F2) and low-scoring (low F2) participants based on median split. Dots represent the number of back-transfer decision for each decision type per each participant. Lines represent mean values per group, and boxes their standard errors

We hypothesised that offenders scoring high on Factor 1 would adhere less to the fairness norm than non-offenders. To test this, we first compared odds ratios (ORs) of choosing a fair rather than an unfair decision between the two groups, and second analysed the association between these odds-ratios with PCL-R Factor 1 and 2 scores in the offender sample. The OR for choosing a fair rather than an unfair decision was lower in the offender group (OR = 0.10, 95% CI = [0.06, 0.16]) than in the non-offenders (OR = 0.24, 95% CI = [0.15, 0.39]), χ^2^ (1) = 6.54, *p* = 0.01, see Fig. 2. The lower the OR, the lower the likelihood is to make a fair back-transfer. Offenders adhered to the fairness norm (and thus behaved inequity averse) only in 12.6% of the trials, compared with 21.96% of the trials in the non-offenders. Within the offender group, Factor 2 was negatively associated with ORs for choosing a fair decision, χ^2^ (1) = 6.92, *p* = 0.01, OR = 0.88 (95% CI = [0.80, 0.97]), but not Factor 1, χ^2^ (1) = 0.53, *p* = 0.47, OR = 0.95 (95% CI = [0.82, 1.10]). Thus higher Factor 2 scores were associated with a lower likelihood to make a fair back-transfer. Offenders scoring high on Factor 2 (median split) adhered only in 7.5% of the trials to the fairness norm, while it was 19.17% of the trials in offenders scoring low on Factor 2. This is against our hypothesis, as we expected norm adherence to be negatively associated with Factor 1 in the offenders.

We further hypothesised that the offenders scoring high on Factor 1 would adhere less to the fairness norm in order to maximise their own profits, thus using a self-advantageous decision more often than a fair decision compared with the non-offenders. To test this, we first compared ORs of choosing self-advantageous rather than fair decision between groups, and second analysed the association between these ORs with PCL-R Factor 1 and 2 scores in the offender sample. The OR for choosing a self-advantageous rather than a fair decision was higher in the offenders (OR = 2.14, 95% CI = [1.16, 3.96]) than in the non-offenders (OR = 0.80, 95% CI = [0.44, 1.47]), χ^2^ (1) = 4.50, *p* = 0.03. Within the offender group, Factor 2 was positively associated with the likelihood of using a self-advantageous rather than a fair decision, χ^2^ (1) = 4.47, *p* = 0.03, OR = 1.19 (95% CI = [1.01, 1.40]), but not Factor 1, χ^2^ (1) = 0.29, *p* = .59, OR = 1.07 (95% CI = [0.84, 1.34]). Thus again against our hypothesis, increased self-advantageous decisions were not associated with Factor 1, but rather with Factor 2, the lifestyle/anti-social subscale.

To further characterise the offenders back-transfer behaviour, we also tested whether there were group differences when contrasting the ORs for choosing self-advantageous and other-advantageous decisions, as well as other-advantageous and fair decisions. The ORs for choosing a self-advantageous rather than an other-advantageous decision were not different between the groups, χ^2^ (1) = 0.04, *p* = 0.83. However, the ORs for choosing an other-advantageous decision compared with a fair decision differed between the groups, χ^2^ (1) = 6.16, *p* = 0.01. The offenders had a higher OR (7.59, 95% CI = [4.59, 12.56]) than the non-offenders (OR = 3.07, 95% CI = [1.90, 4.95]). We followed this up with an analysis of the association between the ORs for choosing an other-advantageous rather than a fair decision and PCL-R Factor 1 and 2 scores in the offender sample. Within the offender group, the ORs were positively associated with Factor 2, χ^2^ (1) = 7.05, *p* = 0.01, OR = 1.14 (95% CI = [1.03, 1.25]), but not with Factor 1, χ^2^ (1) = 0.37, *p* = 0.54, OR = 1.05 (95% CI = [0.91, 1.21]). Thus offenders were not only more likely to make a self-advantageous but also an other-advantageous rather than a fair decision, and both were positively associated with Factor 2. However, since the ORs of choosing a self-advantageous vs. other-advantageous decision did not differ between the groups, the OR differences in self-advantageous and other-advantageous decisions seem to be driven by lower fair decision likelihoods in the offenders, and not increased self-advantageous or other-advantageous decision likelihoods.

**Fig. 3:**
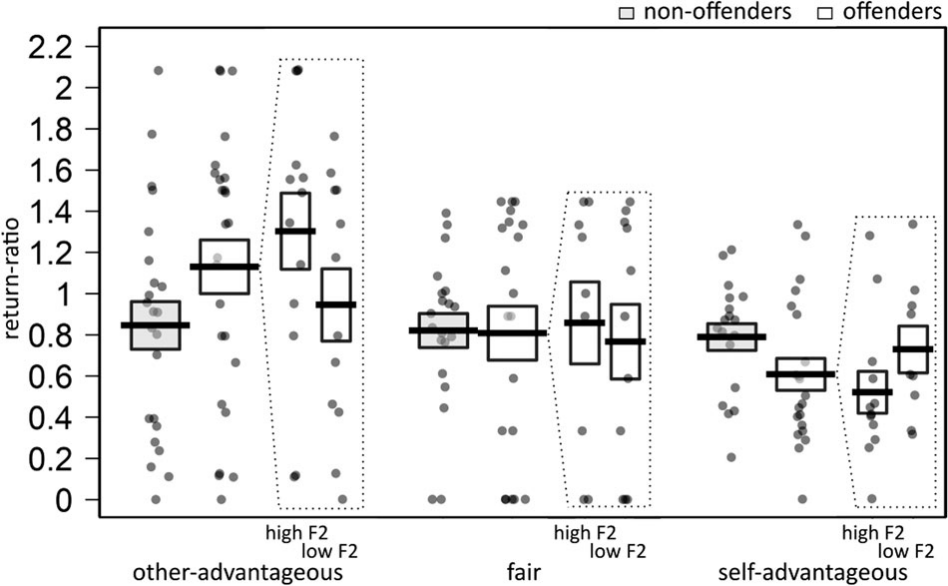
Self-advantageous return-ratios were lower than fair and other-advantageous return-ratios in the offenders, and this was negatively associated with Factor 2 scores. To visualise effects of Factor 2, we categorised offenders into high-scoring (high F2) and low-scoring (low F2) participants, based on median split. Dots represent average return-ratio for every participant, per decision. Lines represent mean values per group, and boxes their standard errors

Part of being profit-maximising is that the actual back-transfers during a self-advantageous decision are as low as possible. Therefore, we hypothesised that the self-advantageous return-ratios are negatively associated with Factor 1 in the offenders. We tested this by first comparing return-ratios between the two groups, the decision types and their interaction, and second by analysing the association between the return-ratios and PCL-R Factor 1 and 2 scores, the back-transfer decision types and their interactions in the offender sample. The return-ratios differed significantly for the three decision types (main effect back-transfer decision: χ^2^ (2) = 12.61, *p* < 0.01), but not for the groups (χ^2^ (1) < 0.001, *p* = 0.98). Importantly, however, there was a significant back-transfer decision x group interaction, χ^2^ (2) = 8.84, *p* = 0.01, see Fig. 3. Decomposing this interaction, we found that the return-ratios did not differ overall for back-transfer decisions between the groups (all *p* > 0.1), and also not in the non-offenders (all *t* < 0.81, *p* > 0.90). In the offenders, however, return-ratios were lower for self-advantageous decisions than for other-advantageous decisions (*t*(43.32) = 4.60, *p* < 0.01), and also compared with fair decisions (*t*(47.24) = 2.82, *p* = .04). Return-ratios did not differ between other-advantageous and fair decisions (*t*(47.46) = 2.37, *p* = 0.11). Within the offender group, there was a significant back-transfer decision x Factor 2 interaction, χ^2^ (2) = 8.35, *p* = 0.02, but no back-transfer decision x Factor 1 interaction, χ^2^ (2) = 0.97, *p* = 0.61. Decomposing the back-transfer decision x Factor 2 interaction, we found that only self-advantageous return-ratios were negatively associated with Factor 2, b = −0.04 (95% CI = [−0.07, −0.02]), *p* < 0.01, but neither the other-advantageous (b = 0.03, 95% CI = [−0.03, 0.09]) nor the fair return-ratios (b = −0.03, 95% CI = [−0.08, 0.03]) were associated with Factor 2, both *p* > 0.31. Thus, the offenders’ lower self-advantageous return-ratios were negatively associated with Factor 2 scores.

Since the offenders displayed only mixed profit-maximising behaviour, they did not earn more money than the non-offenders at the end of the game, F(1, 46) = 0.42, *p* = 0.52 (m_offenders_ = 357.12 MU (SD = 52.84) vs m_non-offenders _= 347.61 MU (SD = 48.84)). The total earnings were also neither associated with Factor 1 (F(1, 22) = 1.24, *p* = 0.28), nor with Factor 2 (F(1, 22) = 1.08, *p* = 0.31).

Our final analysis addressed our hypothesis of increased SCRs in anticipation and during receipt of self-advantageous decisions in offenders scoring high on Factor 1. We tested this by first comparing SCR anticipation between the groups, the back-transfer decision types and their interaction, and second by analysing the association between SCR anticipation and PCL-R Factor 1 and 2 scores, the back-transfer decision types, as well as their interactions. The same analyses were performed with SCR outcome as dependent variable. First, for SCR anticipation, there was a main effect of group (χ^2^ (1) = 4.13, *p* = 0.04), with higher SCR in offenders than non-offenders, but no main effect for back-transfer decision (χ^2^ (2) = 0.59, *p* = 0.75). Importantly, there was a significant back-transfer decision x group interaction, χ^2^ (2) = 10.79, *p* < 0.01. Anticipatory SCR to self-advantageous decisions were higher in the offenders than in the non-offenders, *t*(45.40) = 2.35, *p* = 0.05 (b = −0.94, 95% CI = [−1.71, −0.17]). Surprisingly, anticipatory SCR for a fair decision were also marginally larger in the offenders than in the non-offenders, *t*(44.91) = 2.26, *p* = 0.06 (b = −0.94, 95% CI = [−1.75, −0.14]). Anticipatory SCR for an other-advantageous decision did not differ between the groups, *t*(43.17) = 0.90, *p* = 0.58 (b = −0.33, 95% CI = [−1.03, 0.38]). Within the offender group, there were no anticipatory SCR differences depending on Factor 1 (χ^2^ (2) = 0.27, *p* = 0.87), or Factor 2 (χ^2^ (2) = 1.51, *p* = 0.47), but there was a main effect of back-transfer decision, χ^2^ (2) = 7.80, *p* = 0.02, mirroring the decomposed interaction pattern described above. Thus higher anticipatory arousal to self-advantageous decisions in offenders were not associated with psychopathy scores.

SCR outcome neither differed for back-transfer decisions (χ^2^ (2) = 1.86, *p* = 0.39) nor for group (χ^2^ (1) = 1.51, *p* = 0.22), but there was a significant back-transfer decision x group interaction (χ^2^ (2) = 7.34, *p* = 0.03). However, post hoc *t* tests did not reveal any significant group differences for the three back-transfer decisions. There was only a trend for higher SCR outcome to self-advantageous decisions in the offenders than in the non-offenders, *t*(46.35) = 1.93, *p* = 0.11 (all other *t* < 0.89). Within the offender group, there were also no SCR outcome differences for the three back-transfer decisions associated with Factor 1 (χ^2^ (2) = 0.36, *p* = 0.84), or Factor 2 (χ^2^ (2) = 1.05, *p* = 0.59). However, there was a main effect of back-transfer decision, χ^2^ (2) = 6.35, *p* = 0.04, mirroring the decomposed interaction pattern described above. Thus, SCR outcome did not decisively reveal increased arousal to self-advantageous decisions in offenders.

## Discussion

In this study, we investigated whether the personal/affective dimension of psychopathy (Factor 1) is associated with fairness norm violations during a repeated trust game, and whether these offenders prefer to maximise their own profit because of increased reward sensitivity^4,6^. Offenders indeed violated fairness norms more frequently and were more likely to make self-advantageous decisions with lower return-ratios than non-offenders. However, these findings were not positively associated with Factor 1, but rather with Factor 2 scores in the offenders. Factor 2 comprises the lifestyle (impulsive and irresponsible behaviour) and anti-social dimension of the PCL-R^36^. Our hypothesis of hyperresponsive arousal to reward in the psychopathic offenders was not supported, as there was only a general increase in SCR in anticipation of self-advantageous decisions for all offenders. Surprisingly, the norm-violating behaviour in the offenders scoring high on Factor 2 did not lead to different total earnings compared with the non-offenders.

Offenders scoring high on Factor 2, hence called anti-social offenders, of the PCL-R are characterised as being easily bored, impulsive and having a long history of anti-social behaviour even before any conviction^3^. In prison, they break rules and are non-compliant during treatment^37^, and are also more likely to commit violent crimes again in the future than offenders scoring high on Factor 1 of the PCL-R^38^. Our results reinforce that extra attention should be paid to the treatment of these anti-social offenders. We not only provide empirical support to the norm violations that forensic clinicians encounter frequently in their treatment programmes^38^ but they can also be a starting point for specialised treatment. It is possible that the norm violations in our sample are related to deficits in spontaneous empathising abilities^39^. Research by Mayer et al.^40^ suggests that these abilities might be trainable, since violent offenders increased their monetary distributions in a dictator game after an empathy enhancement intervention. Future research should establish whether anti-social offenders would decrease their norm violations with a treatment programme that increases their empathic concerns.

We were unable to demonstrate decisively why anti-social offenders violate fairness norms, as our profit-maximisation hypothesis was only partly supported. The differences in the decision likelihoods of the three back-transfer options were mainly driven by a decreased number of fair decisions, and were not driven by an increased number of self-advantageous decision in the anti-social offenders. In addition, even though the offenders’ anticipatory arousal was reflective of hyperresponsiveness to rewards, this was not related to any psychopathy measures. Only the low self-advantageous return-ratios in the anti-social offenders clearly supported the profit-maximising hypothesis. Therefore, we conclude that, while relevant, profit-maximisation might not be the main driver of anti-social offenders´ behaviour in situations with repeated, anonymous interactions. The current experimental situation provided only limited external validity, and subjects were only able to gain a comparably low amount of money. Additional research is needed to determine the translatability of our findings to criminal behaviour in anti-social offenders.

It is conceivable that the anti-social offenders´ norm violations were motivated by a different behavioural strategy, which we were unable to detect with our paradigm. Indeed, their most frequent back-transfer decision was other-advantageous, which suggests that they were prosocial towards their interaction partner. However, according to current theorising, psychopathic offenders lack many of the prerequisites that induce a concern for prosociality and would facilitate behaviour that benefits others^41^. They show deficits in affective empathy^23,39,42–44^, display little shame and guilt^45^ and make deviant moral decisions^46^. Therefore, we suggest that their frequent other-advantageous decisions were not based on other-related concern, but were rather part of a manipulative strategy that benefitted them in the long run. Indeed, in the literature, a manipulative nature is widely regarded as a core feature of psychopathy^47^. Even though we were unable to measure a manipulative strategy in our task, the intention of the anti-social offenders might have still been to manipulate the investor. After all, their norm violations and low self-advantageous back-transfers did not have any repercussions, as they earned the same amount of money as the non-offenders. Notably, we are neither making any assumptions nor do we have data to show whether these strategies operated intentionally and explicitly. Other decision-making paradigms that investigate non-prosocial cooperative strategies^41^ will help understand psychopathic offenders’ motivation and strategies during repeated interactions. The anti-social offenders’ other-advantageous behaviour in our study might be a case of by-product reciprocity of otherwise selfish intentions. However, this remains to be tested in future studies.

Our arousal findings only partially support Arnett´s notion of hyperresponsiveness to rewards in psychopathic offenders^20^. Even though we did find increased anticipatory arousal to self-advantageous decisions in the offenders, this was not associated with any psychopathy measure. Studies on empathy have found similar patterns of arousal discrepancies between offenders and non-offenders, which were not specific to any psychopathy measure^23,24,48,49^. However, the psychopathy scores of our offenders are (similar to other European offender samples) lower than those of American psychopathic offenders. We cannot exclude that with a larger variation between high-scoring and low-scoring psychopathic individuals, arousal patterns might diverge between these groups. We believe that our rather unspecific results are reflective of a general effect of hyperresponsiveness to rewards in violent offenders, but this needs replication in samples with more extreme psychopathy scores as the ones present in the sample to which we had access to.

We used two different psychopathy measures in our study, since we tested two different cohorts (a community and an offender sample). As a result, we could not directly compare how the degree of psychopathy differently affected the normative behaviour in the offenders and the non-offenders. Our study was also not designed to test this, as we only recruited individuals scoring low on psychopathy and with little intersubject variance in the community sample. Nonetheless future studies should directly compare decision-making behaviour in psychopathic individuals from a community sample with an offender sample, as the decision-making literature points to vastly different behaviour between them. Non-delinquent psychopathic individuals only rarely abide to norms^50–53^ and punish those who deviate from social norms to a much lesser degree than incarcerated psychopaths^51,54,55^. This might be due to a distorted perception of offer fairness, as participants from a community sample scoring high on psychopathy rated unfair offers as more fair than those scoring low on psychopathy^56^. In a recent study by Gong et al.^57^, non-delinquent psychopathic individuals behaved much more profit-maximising than the psychopathic offenders in our study. The non-delinquent psychopathic individuals ignored their interaction partners’ expectations and felt less guilty when they violate them than non-delinquent subjects scoring low on psychopathy^57^. This highlights the necessity that future work compares participants scoring high on psychopathy from a prison cohort with those from a community cohort to establish similarities and differences in norm adherence behaviour during repeated interaction decision-making paradigms.

With the present work, we aim to set a first step in characterising anti-social offenders’ behaviour during repeated sequential decision-making interactions, by establishing that they do not adhere to social norms and that this is only minorly driven by profit-maximising back-transfer behaviour. Repeated decision-making paradigms enable psychopathic offenders to display complex, strategic behaviour, which creates space for researchers to study how and why psychopathic offenders manipulate their interaction partners. We propose that future studies employ non-prosocial cooperative decision-making paradigms to specifically target manipulative strategies in psychopathic offenders. In addition, we suggest treatment programmes targeting empathic abilities particularly in anti-social offenders, since even in standardised experimental settings anti-social offenders violate norms which might extrapolate to criminal behaviour in real life. Overall, our study outlines a promising new path for advancing our understanding of the norm-violating nature of (anti-social) psychopathic offenders.

## Supplementary information

Supplementary Material
