# Supplementary material for: Fairness norm violations in anti-social psychopathic offenders in a repeated trust game
Source: Transl Psychiatry. 2019 Oct 21;9:266. doi: 10.1038/s41398-019-0606-3 (PMC6803633; doi:10.1038/s41398-019-0606-3)
Supplement: Supplementary file 1 — Supplementary Material [file 41398_2019_606_MOESM1_ESM.docx]

**Supplementary Material to**

**Fairness norm violations in anti-social psychopathic offenders in a repeated trust game**

Lisa A. Rosenberger, Daniela M. Pfabigan, Benjamin Lehner, Katinka Keckeis, Eva-Maria Seidel, Christoph Eisenegger and Claus Lamm

**Supplementary Methods**

Repeated trust game pre-programmed investment decisions

In general, investments in the following round increased when the participant made a high back-transfer. The investments changed in the following ways: for extreme negative back-transfers, where the participant did not make a back-transfer, investments in the next round were low and ranged from 2 to 4 MU. In case that the participants did not make a back-transfer in the entire task, the investments in the last two rounds increased to 9 and 12 MU to coax the participant into making a back-transfer. However neither of the participants displayed such behaviour. For negative back-transfers, where the participant´s back-transfer was lower than the investment, investments in the next round were low as well and ranged from 2 to 5 MU. To make the investment behaviour more realistic (and less static) we randomly interspersed these trials with noisy trials where investments ranged from 9 MU to 14 MU. For positive back-transfers, where the back-transfer was larger than the investment, investments in the next round were also positive and ranged from 6 and 10 MU. Noise trials were randomly interspersed with investments of either 4 MU, 5 MU, 15 MU, or 16 MU. Extremely positive back-transfers, which were more than twice the size of the investment, were followed by extremely positive investments in the next round, with a range between 14 and 18 MU. Noise trials comprised investments of either 5 MU, 9 MU, or 10 MU.

Questionnaires

*Psychopathy assessment offenders*

The offender sample´s degree of psychopathy was assessed with the Psychopathy Checklist – Revised ^1^. The scale comprises a 20-item semi-structured interview consisting of a questionnaire (items are scored on a scale from 0 (*absent*) to 2 (*present*)) and a review of the offenders’ records. Total scores can range from 0 to 40. Our samples average total score is comparable to other Austrian psychopathic offender samples (N = 1046, m = 20.3, SD = 7.6) ^2^. Besides the total score, we also assessed the influence of Factor 1 and Factor 2 subscales on norm adherence and arousal in our study. Factor 1 comprises items from the interpersonal and affective subscale, whereas Factor 2 captures items from the lifestyle and antisocial subscales ^1^.

*Psychopathy assessment non-offenders*

In the non-offenders we assessed psychopathy with the German version of the Psychopathic Personality Inventory – revised PPI-R, ^3^. The self-administered scale contains 154 items, which were scored on a 4-point scale ranging from *false* to *true*. Each item corresponds to one of nine scales, which are grouped into a three factor model ^4^. Our non-offender group´s average total score is lower than in a German male student sample (N = 148, m = 325.42, SD = 24.92) and a German male offender sample (N = 57, m = 338.71, SD = 25.68) ^5^. Therefore, we can conclude that our non-offenders did not display a high degree of psychopathy.

*Impulsiveness assessment*

Psychopathy is tightly interconnected with impulse-control problems (see Poythress *et al.* ^6^ for a discussion). Both the PCL-R and the PPI-R questionnaires have items that specifically assess impulsivity, and in both scales Factor 2 specifically captures the erratic and reactive side of psychopathy. To control for differences in normative behaviour as a result of impulsivity differences in the samples, we included the German version of the 11th revision of the Barratt Impulsiveness Scale (BIS-11) ^7^ in our analyses as a covariate of no-interest. It is a self-report instrument comprising 30 items with answers ranging from *rarely/never* to *almost always/always*. High scores signify high impulsivity. Each item corresponds to one of three factors: attentional, motor, and non-planning impulsivity.

*Intelligence Assessment*

We matched the non-offenders with the offender samples´ intelligence scores. For this we used Raven’s Standard Progressive Matrices (SPM) ^8^. This is a Rasch homogenous, language-free intelligence test for the assessment of non-verbal reasoning. The long version of the SPM is divided into five sets, each including 12 items. For each item a participant has to determine which of several alternative parts fits into a pattern. The offenders at the correctional facility were all routinely assessed with a short version of the SPM, which only includes 32 items.

*Trust assessment*

The German version of the Interpersonal Trust Scale (ITS) ^9^ is a unipolar scale consisting of 17 items measuring interpersonal trust. Items are scored on a 5-point Likert scale ranging from *strongly disagree* to *strongly agree*. High scores signify high interpersonal trust.

Statistical analyses

Data were analysed in R (version 3.4.2 ^10^) with the lme4 package version 1.1.14 ^11^. The reported chi-square tests are calculated with type 3 sum of squares with the car package version 3.0 ^12^. Post-hoc tests for interactions only including factorial variables were performed with the emmeans package version 1.2.3 ^13^ and p-values for multiple comparisons were Tukey-adjusted. The reported odds ratios serve as effect sizes. Post-hoc comparisons for interactions including continuous variables were performed with the SjPlot package version 2.4.1 ^14^ where p-value estimations were based on Wald z-statistics. Here, the reported betas serve as effect sizes. All the questionnaire scores were mean-centred. Including these predictors increased the fit of the models (as measured with the BIC). In the cases where the investor made investments lower than 6 MU the back-transfer decision options were limited, as no fair (with investments ≤ 4 MU) or self-advantageous (with investments ≤ 5 MU) decisions were possible. Our results do not change when excluding investments smaller than 6 (see ^15^ for results).

Post-hoc power analyses with Westfall’s online calculator^16^ for our primary group comparison of fair over unfair back-transfer decisions resulted in a power of 0.815. Analyses are based on a 2-group between participants design with random factor for participants, with a Cohen’s d of 0.51 (transformed log odds-ratio of 0.931 of group effect), 20 observations per participant, 24 participants per group, and the pre-defined variances.

References

1. Hare R (2003). Manual for the Revised Psychopathy Manual for the Revised Psychopathy Checklist (2nd edn): Multi-Health Systems.

2. Neumann CS, Hare RD, Pardini DA. Antisociality and the Construct of Psychopathy: Data From Across the Globe. *J Pers* 2015; **83**(6)**:** 678-692.

3. Alpers GW, Eisenbarth H. *Psychopathic Personality Inventory-Revised (PPI-R), German Version. Manual.* Hogrefe: Göttingen, Germany, 2008.

4. Lilienfeld SO, Widows MR (2005). Psychopathic Personality Inventory - Revised.

5. Eisenbarth H, Alpers GW. Validierung der deutschen Übersetzung des Psychopathy Personality Inventory (PPI). *Z Klin Psychol Psychother* 2007; **36**(3)**:** 216-224.

6. Poythress NG, Hall JR. Psychopathy and impulsivity reconsidered. *Aggr Violent Behav* 2011; **16**(2)**:** 120-134.

7. Preuss UW*, et al*. Psychometric evaluation of the German version of the Barratt Impulsiveness Scale. *Nervenarzt* 2008; **79**(3)**:** 305-319.

8. Raven JC (1969). Standard progressive matrices: sets A, B, C, D, and E. Lewis: London, UK.

9. Amelang M, Gold A, Kuelbel E. Ueber einige Erfahrungen mit einer deutschsprachigen Skala zur Erfassung zwischenmenschlichen Vertrauens (Interpersonal Trust). *Diagnostica* 1984; **30:** 198 - 215.

10. R Core Team (2017). R: A language and environment for statistical computing. R Foundation for Statistical Computing: Vienna, Austria.

11. Bates DM, Mächler M, Bolker B, Walker S. Fitting linear mixed-effects models using lme4. *Journal of Statistical Software* 2015; **67**(1)**:** 1 - 48.

12. Fox J, Weisberg S. *An {R} Companion to Applied Regression*. Sage: Thousand Oaks CA, 2011.

13. Lenth RV (2018). emmeans: Estimated Marginal Means, aka Least-Squares Means.

14. Lüdecke D (2018). sjPlot: Data Visualization for Statistics in Social Science.

15. Rosenberger LA (2019). Analyses scripts to manuscript "Fairness norm violations in anti-social psychopathic offenders in a repeated trust game". : <https://osf.io/x5sav>.

16. Westfall J. PANGEA: Power ANalysis for GEneral Anova designs. *Working paper* 2016.
